# Development of Negative-Temperature Cement Emulsified Asphalt Spraying Materials Based on Spraying Performance and Rheological Parameters

**DOI:** 10.3390/ma17133137

**Published:** 2024-06-26

**Authors:** Yitong Hou, Kaimin Niu, Bo Tian, Junli Chen, Xueyang Li

**Affiliations:** 1 School of Civil Engineering, Chongqing Jiaotong University, Chongqing 400074, China; 2 Research Institute of Highway Ministry of Transport, Beijing 100088, China; 3 Chongqing Communications Construction (Group) Co., Ltd., Chongqing 401120, China

**Keywords:** negative-temperature environment, cement emulsified asphalt, spray performance, rheological properties, frost resistance, pore structure

## Abstract

To develop a cement emulsified asphalt composite (CEAC) that can be sprayed under a plateau negative temperature environment, the effects of the water–solid ratio, calcium aluminate cement substitution rate, emulsified asphalt content, sand–binder ratio, and polyvinyl alcohol (PVA) fiber content on the spraying performance and rheological parameters of CEAC were explored through the controlled variable method. Additionally, the correlation between the spraying performance and rheological parameters of CEAC was established, and the optimal proportion of CEAC was determined. Then, the difference in frost resistance and pore structure between the cement slurry (CS) without emulsified asphalt and CEAC at the optimum proportion was analyzed. The results showed that the optimum proportions for sprayed CEAC were 0.14 water–solid ratio, 0.5 sand–binder ratio, 25% substitution of calcium aluminate cement, 5% emulsified asphalt content, and 1.5% PVA fiber volume mixing. The yield stress and plastic viscosity of CEAC were positively correlated with the build-up thickness, whereas the rebound rate and the latter showed a negative correlation. The spraying performance may be described by the rheological parameters; the ranges of yield stress and plastic viscosity of 2.37–3.95 Pa·s and 77.42–108.58 Pa, respectively, produced the best spray ability. After undergoing an equivalent number of freeze–thaw cycles, CEAC exhibited lower mass and strength loss rates compared to CS, thereby demonstrating superior frost resistance. In addition, the pore structure analysis showed that the difference in capillary and macropore contents was the main reason for the variability in frost resistance between CS and CEAC.

## 1. Introduction

One type of quick-setting, hardened concrete is called shotcrete, for which a pressure gun is used to spray fine stone concrete on a target surface; it is widely used in tunnel lining structure support, a variety of repair and reinforcement projects, with good economic efficiency, short construction period, and so on, and the combination of reinforcement mesh, anchors, and other support methods can significantly improve the protective capacity of a structure [1,2,3]. The application technologies of shotcrete mainly include the dry-mix process and wet-mix process [4]. As the wet-mix process has better homogeneity and durability, and may successfully lower the dust concentration and rebound amount, the wet-mix process has become the development trend of shotcrete technology in the world [5,6,7]. However, due to the need to mix the aggregate, cement, and water in the designed proportion before construction, it is not possible to adequately ensure the quality of construction under adverse weather conditions, which limits this technique’s application in the permafrost regions of high-altitude and cold regions [8]. Among the factors that influence shotcrete’s performance, the properties of the binder hold the utmost significance [9]. Therefore, there is a need to develop high-quality sprayed materials suitable for permafrost regions. Although some scholars have successfully applied wet-sprayed concrete to high-altitude permafrost regions with negative temperatures all year round as well as to tunnels in cold regions by adding additives such as early-strength antifreeze binders, antifreeze, and accelerators to shotcrete, its performance is not effectively guaranteed [10,11,12]. In addition, due to the frequent alternation of positive and negative temperatures in perennial permafrost regions and tunnel entrance sections in cold regions, the internal moisture of shotcrete expands by freezing and thawing, so the frost resistance of shotcrete is also an important indicator of its durability performance [13]. Some academics have also conducted in-depth research on the resistance of shotcrete to frost. Haldane [14] believes that the frost resistance of shotcrete is influenced by the distribution of pores and is intricately linked to the water–cement ratio. Wan et al. [15] discovered that incorporating micro- and nano-bubbled water into shotcrete can improve its pore structure, thus improving the resistance of shotcrete to frost. Hu et al. [16] performed an investigation into the current status of shotcrete research in cold tunnels, summarized the various factors that influence shotcrete tunnels situated in cold regions, and forecasted the future trends in the development of such tunnels. Holter et al. [17], in response to the characteristics of shotcrete in a cold-region tunnel lining that is susceptible to freezing damage, investigated the freezing resistance of shotcrete employing a specific ratio of water to cement of 0.45–0.47 through experiments and put forward a new functional freeze–thaw cycle test method, which can more realistically simulate the conditions of moisture content and freezing gradients within a tunnel lining. Kalhori et al. [18] examined the influence of nanomaterials on the frost resistance properties of shotcrete, revealing that both nanoclay and nanosilica enhance its frost resistance capabilities. Furthermore, the study indicated that nanosilica exhibits a superior improvement effect compared to nanoclay.

Rheology pertains to the scientific discipline that investigates the deformation and flow behaviors of substances in response to external forces. For cement-based materials, which are non-homogeneous, multi-phase, and non-linear, their deformation and destructive behaviors under external forces are affected by a variety of factors. In cement-based materials, rheology mainly studies their deformation and flow characteristics, including properties such as fluidity, plastic viscosity, yield stress, creep, and relaxation. These characteristics play a crucial role in determining the spraying efficiency of cement-based materials. Yun et al. [19] conducted a study exploring the impact of mineral admixtures on the rheological properties of shotcrete by using Bingham’s model, and based on the rheological properties, suggested a novel technique aimed at enhancing the pumping efficiency and spraying capabilities of shotcrete. Pan et al. [20] examined the rebound rate of sprayed concrete in relation to its rheological characteristics, including slump, yield stress, plastic viscosity, and others, discovering that these properties hold significant importance in the performance of shotcrete. Their findings revealed that variations in slump, yield stress, and water secretion rate exert a considerable influence on the rebound rate of concrete. Furthermore, they derived a multiple linear regression equation that can be utilized to predict the rebound rate. Feng et al. [21] formulated a sprayable ultra-high ductility magnesium cementitious composite (UHDMC) and established a correlation between sprayability factors and rheological parameters. This correlation enabled the determination of the optimal mixing ratio suitable for spraying UHDMC. In conclusion, to better predict the spraying performance of cementitious sprayed materials, the relationship between their sprayability and rheological properties needs to be investigated.

**Figure 1:**
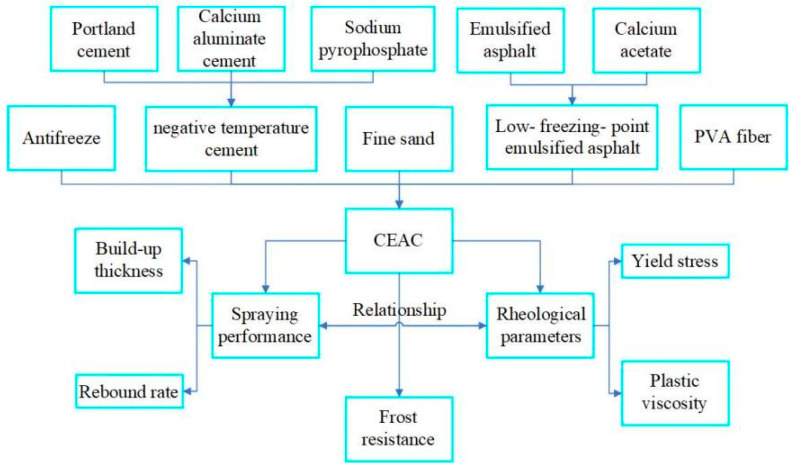
Experimental flowchart.

Based on this, in order to achieve shotcrete construction in negative-temperature environments and improve the frost resistance of concrete, the present study endeavors to devise a cement emulsified asphalt composite (CEAC) suitable for spraying in cold environments. Furthermore, it aims to explore the influence of various factors, including water–solid ratio, sand–binder ratio, calcium aluminate cement substitution rate, emulsified asphalt dosage, and PVA fiber dosage, on the sprayability characteristics and rheological parameters of CEAC. Moreover, utilizing identical mix parameters, this study conducted an analysis of data fitting to investigate the correlation between sprayability characteristics and rheological parameters, ultimately determining the optimal mix ratio. Finally, the difference in frost resistance and pore structure between cement slurry (CS) without emulsified asphalt and CEAC at the optimal mix ratio was determined by the MIP test, which revealed the intrinsic reasons for the difference in frost resistance. The results provide guidance for the application of CEAC in tunnels and other spraying projects in high-altitude permafrost regions. The flowchart of this study is shown in Figure 1.

## 2. Experimental Program

### 2.1. Materials

**Figure 2:**
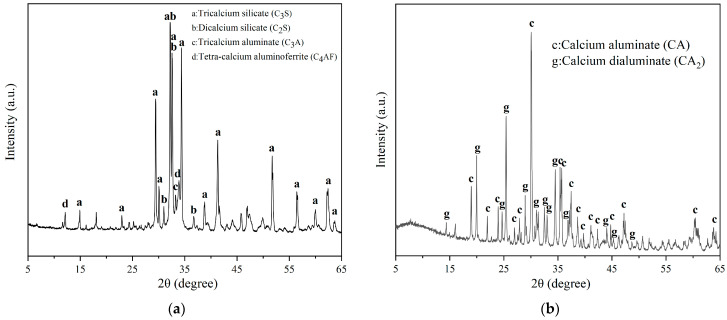
XRD pattern of the cement: (**a**) Portland cement and (**b**) calcium aluminate cement.

According to the results of previous studies by our group, a mixture of ordinary silicate cement and calcium aluminate cement was used as the negative-temperature cement, and the antifreeze used was a 20 wt.% concentration of sodium nitrite solution, with 3% of the total cement mass of sodium pyrophosphate used as the accelerator [22]. Low-freezing-point emulsified asphalt was prepared by adding 8% calcium acetate by mass to emulsified asphalt, which was externally mixed. Two kinds of cement were provided by a cement factory in Beijing. The compositional and XRD patterns of the cement are shown in Table 1 and Figure 2. The emulsified asphalt used was obtained from Chongqing CNOOC Modern Transportation Oil Material Co., Ltd. (Chongqing, China). The sodium pyrophosphate, sodium nitrite, and calcium acetate were AR reagents produced by Shanghai Aladdin Biochemical Science and Technology Co., Ltd. (Shanghai, China). The water used was tap water.

Quartz sand with a diameter of 0–0.1 mm was selected for the fine sand, which was provided by Beijing Construction Engineering Group Co., Ltd. (Beijing, China). The fiber material used was PVA fiber produced by Changzhou Tianyi Engineering Fiber Co., Ltd. (Changzhou, China). The performance factors of the PVA fiber are shown in Table 2. The water used was tap water.

### 2.2. Mixing Ratio of CEAC

To obtain the optimum mixing ratio of CEAC, the control variable method was utilized to first determine the optimum dosing conditions for the water–solid ratio (W/S) and sand–binder ratio (S/B). The effects of the calcium aluminate cement substitution rate (CAC) and emulsified asphalt content (EA) on the CEAC spraying effect were subsequently studied. Finally, the effect of the volume content of PVA fibers (PVA) on the effect of CEAC spraying was studied. Based on the above factors, the mix ratios were optimized step by step to obtain the best mix ratios, and the test mix ratios of CEAC are listed in Table 3, where W/S, S/B, CAC, EA, and PVA stand for the water–solid ratio, sand–binder ratio, substitution rate of calcium aluminate cement, content of emulsified asphalt, and volumetric percentage of PVA fibers, respectively, and the other parameters are fixed when one of them is changed.

### 2.3. Experiment Methods

#### 2.3.1. Spraying Test

**Figure 3:**
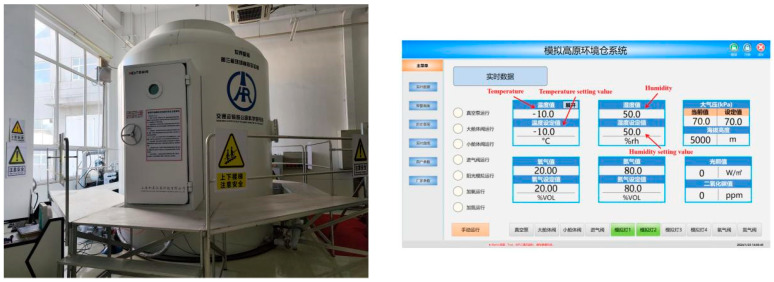
Plateau environment simulation silo.

**Figure 4:**
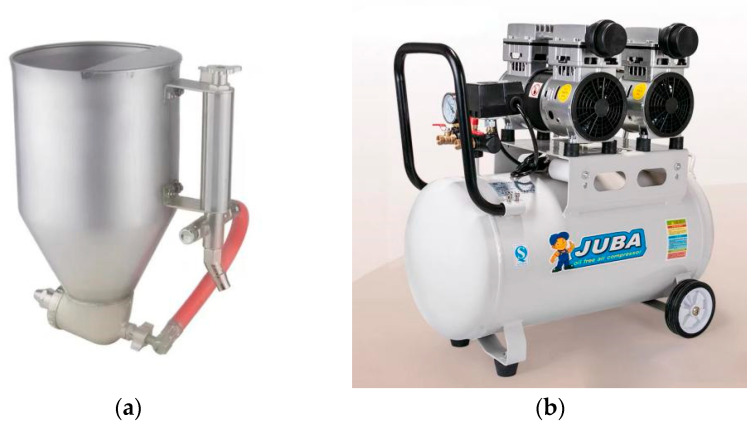
Spraying equipment. (**a**) Spraying device. (**b**) Air compressor.

**Figure 5:**
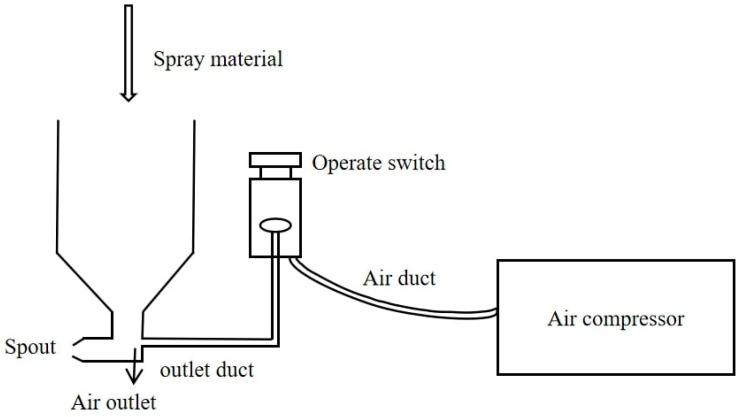
Spraying principle of the spraying equipment.

To accurately simulate the negative-temperature environment at high altitudes, the spraying test and curing of specimens in this study were carried out in a plateau environment simulation warehouse independently developed by the Highway Research Institute of the Ministry of Transportation and Communications. The ambient temperature was set at −10 °C, the humidity was set at 50%, and the appearance and operation interface of the simulation warehouse are shown in Figure 3. A 9511 paint spray gun produced by the Taizhou Road and Bridge Construction Pneumatic Tool Factory was used for the CEAC spraying test, and the spraying equipment is shown in Figure 4. The spraying principle is shown in Figure 5.

All the raw materials for the test needed to be placed in the environment simulation silo to keep warm for 24 h. First, cement and sand were mixed with dry mixing in a mixer for 120 s. Then, emulsified asphalt was introduced into the mixer and blended for an additional 60 s. Eventually, PVA fibers were incorporated and mixed for 120 s, resulting in the production of the CEAC. The spraying test of CEAC was sprayed according to Chinese industry standard JGJT 372-2016 [23].

#### 2.3.2. Spraying Performance Test

**Figure 6:**
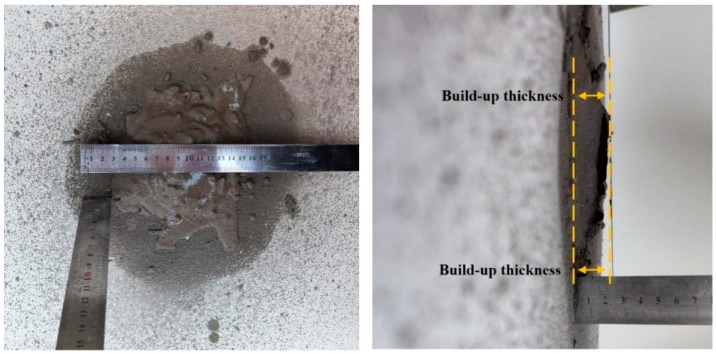
Build-up thickness test.

The spraying performance was assessed based on two key metrics: the build-up thickness and the rebound rate. The build-up thickness serves as a metric indicative of the cement-based materials’ ability to adhere securely to the wall surface. It is measured by spraying continuously at a base point perpendicular to the spraying surface until the material falls off under its own gravity; at this time, the thickness of the material is the build-up thickness, and the method for measuring it is illustrated in Figure 6.

**Figure 7:**
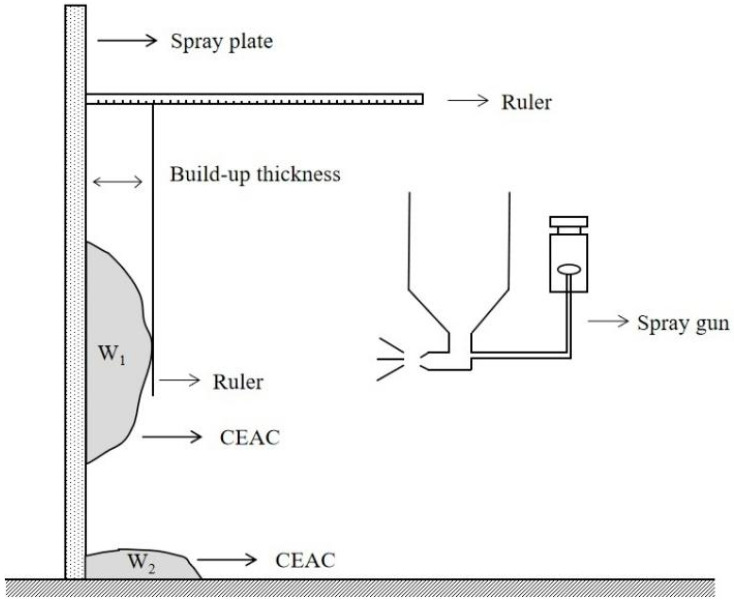
Rebound rate test.

The rebound rate serves as a measure of how well the sprayed material adheres to the wall. A higher rebound rate indicates inferior application of the coating and substandard adhesion properties of the material. Conversely, a lower rebound rate signifies a satisfactory spraying effect and the material’s ability to effectively bond with the sprayed structural surface. The rebound rate test method refers to the Japanese standard JSCE-F 563-2005 [24]; the rebound rate test method is shown in Figure 7, and the calculation formula is presented in Equation (1):

(1) $$R=\frac{W_{2}}{W_{1}{+W}_{2}}\times 100\%$$

where *R* is the rebound rate (%), W_1_ is the mass of CEAC sprayed on the wall (g), and W_2_ is the mass of CEAC dropped on the floor (g).

#### 2.3.3. Rheological Test

**Figure 8:**
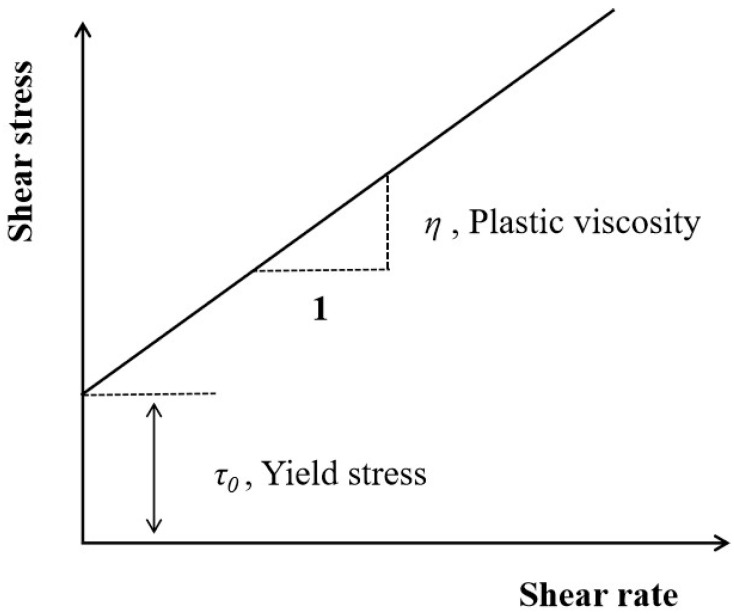
Bingham model.

The Bingham model stands out as the most widely employed model in the rheological examination of cement-based materials, as depicted in Figure 8. According to the Bingham model, when the shear stress is greater than the yield stress, the shear stress of cement-based materials is linearly related to the shear rate. Its intercept is the yield stress, and the slope is the plastic viscosity. In our investigation, the Bingham model was utilized to ascertain the yield stress and plastic viscosity of CEAC [25,26], and the corresponding calculation formula is outlined in Equation (2):

(2) $$\tau =\tau_{0}+\eta \cdot \gamma$$

where *τ* is the shear stress (Pa), *τ*_0_ is the yield stress (Pa), *η* is the plastic viscosity (Pa·s), and *γ* is the shear rate (s^−1^).

**Figure 9:**
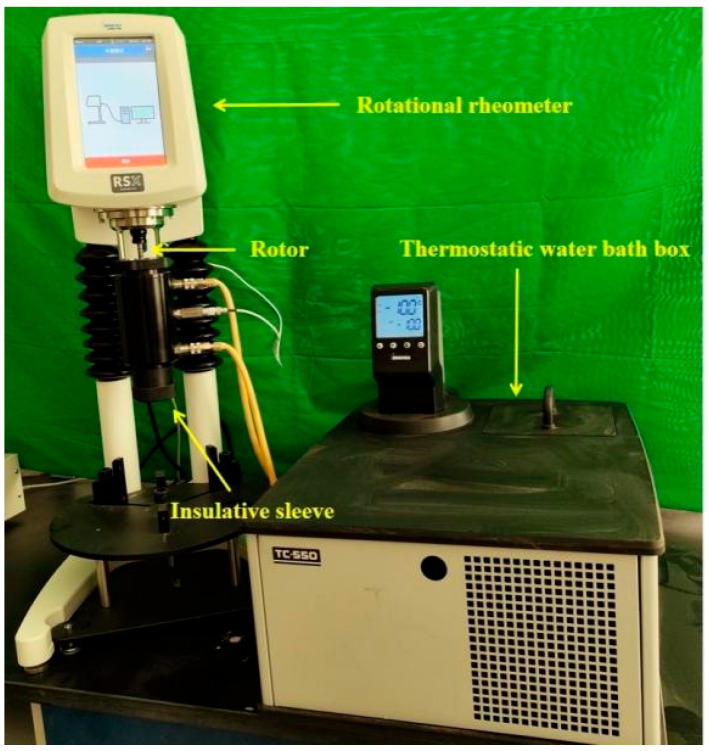
RSX rotational rheometer.

The rheological parameters of the CEAC were tested using an RSX rotational rheometer manufactured by Brookfield Company, as shown in Figure 9. The holding solution was a mixture of ethanol and water, and the test temperature was set at −10 °C. The test was performed using a measuring rotor with a diameter of 40 mm. First, the CEAC to be tested was placed in an insulation sleeve for 30 min. Then, the CEAC mixture was subjected to a rheological test with a shear rate ranging from 0 to 10 s^−1^. The first data point was taken at 1 s^−1^, followed by one data point every 1.5 s^−1^, and the Bingham model was chosen to fit the rheological curves to obtain *τ*_0_ and *η*.

#### 2.3.4. Frost Resistance Test

According to the JGJT 70-2009 [27], CS and CEAC with the best matching ratio were sprayed into a cube mold with a side length of 70.7 mm and then placed in an environment simulation warehouse for negative-temperature maintenance. After undergoing a 28 d curing process with negative-temperature maintenance, the specimens were then subjected to a total of 100 freeze–thaw cycles. Following every 25 cycles of freezing and thawing, the rate of loss in both quality and strength of the specimen was tested.

#### 2.3.5. Mercury Intrusion Porosimetry (MIP) Test

The CS and CEAC specimen blocks, which were cured at a negative temperature until 28 days of age, were cracked and put into an ethanol solution to terminate the hydration, after which the specimen blocks were removed and put into a 60 °C oven to dry for 4 h before the test. The pore structure of the test blocks was tested by a fully automatic mercuric pressure tester (modeled as Autopore IV 9500; manufactured by Mack Corporation, Flagstaff, AZ, USA).

## 3. Results and Discussion

### 3.1. Factors Affecting Spraying Performance and Rheological Parameters

#### 3.1.1. Effect of the Water–Solid Ratio (W/S)

**Figure 10:**
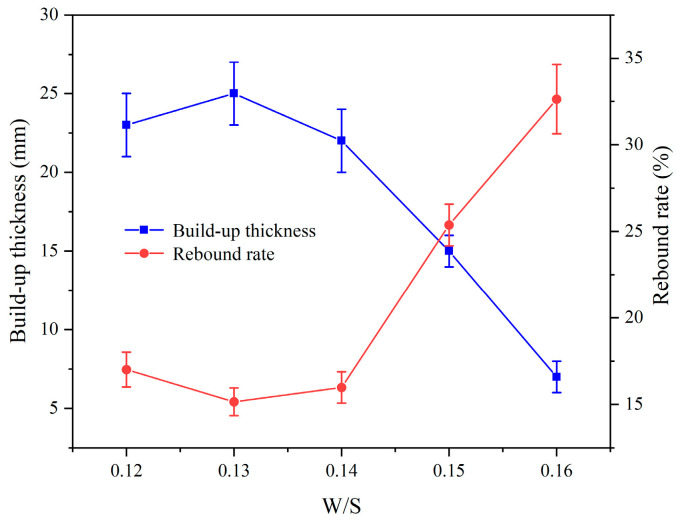
Effect of the W/S on the spraying performance of CEAC.

Figure 10 illustrates the influence of the W/S on the spraying performance of CEAC. As the W/S increased, the build-up thickness of CEAC sprayed in a single application initially rose and subsequently declined, while the rebound rate initially decreased and later increased. Notably, when the W/S reached 0.13, CEAC exhibited optimal spraying performance.

**Figure 11:**
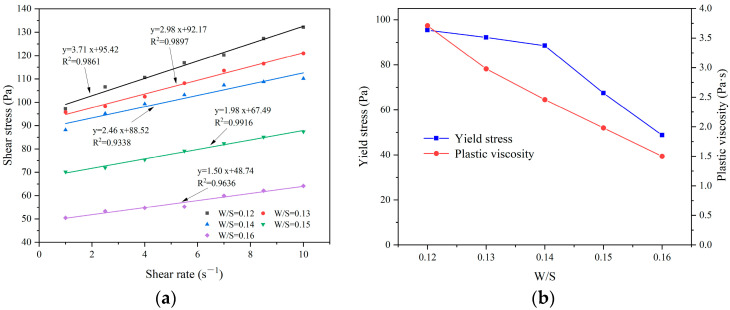
Effect of the W/S on the rheological parameters of CEAC: (**a**) rheological parameter fitting curve and (**b**) yield stress and plastic viscosity.

Figure 11 shows the effect of the water–solid ratio on the rheological properties of CEAC. Figure 11a shows the fitting relationship between shear stress and the shear rate of CEAC at different water–solid ratios. The results showed that there was a good linear relationship between shear rate and shear stress, and R^2^ was between 0.93 and 0.99. Figure 11b demonstrates the impact of the W/S on the rheological parameters of CEAC. As the W/S increased, the yield stress and plastic viscosity of CEAC decreased. Generally, a higher W/S leads to improved rheological properties [28]. However, considering the spraying performance of CEAC, when the W/S exceeded 0.14, the slurry’s fluidity became excessive, resulting in a significant reduction in the build-up thickness. Conversely, at W/S below 0.14, the slurry exhibited excessive viscosity, compromising its workability. Consequently, an optimal W/S of 0.14 was chosen. Under these conditions, CEAC achieved a build-up thickness of 22 mm and a rebound rate of 15.98% after a single spraying treatment, while its yield stress and plastic viscosity were 88.52 Pa and 2.46 Pa·s, respectively.

#### 3.1.2. Effect of the Sand–Binder Ratio (S/B)

**Figure 12:**
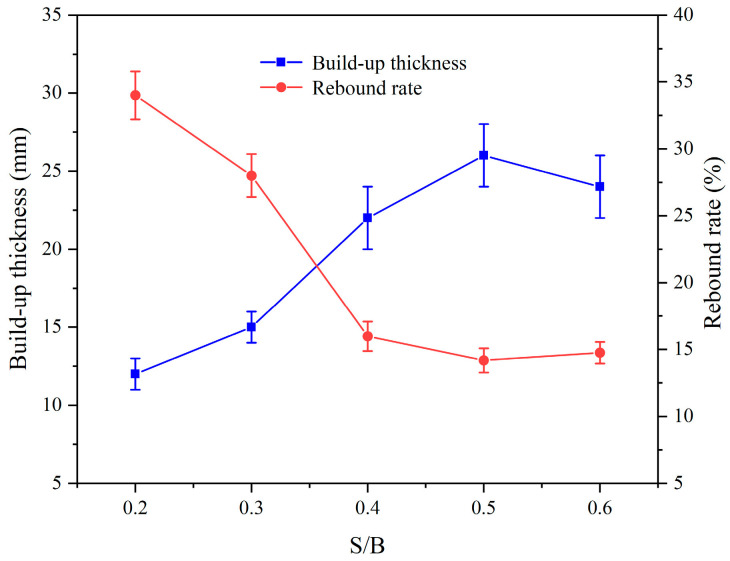
Effect of the S/B on the spraying performance of CEAC.

Figure 12 illustrates the influence of the S/B on the spraying performance of CEAC. As the S/B rose, the build-up thickness of CEAC after a single spraying session initially increased, reaching a peak, and then gradually decreased. Similarly, the rebound rate initially decreased before gradually increasing. Notably, when the S/B was set at 0.5, CEAC exhibited optimal spraying performance. Under these conditions, the build-up thickness measured 26 mm, while the rebound rate stood at 14.18%.

**Figure 13:**
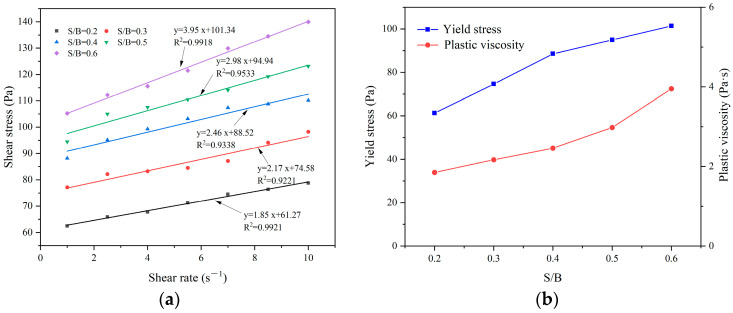
Effect of the S/B on the rheological parameters of CEAC: (**a**) rheological parameter fitting curve and (**b**) yield stress and plastic viscosity.

Figure 13 shows the effect of the sand–binder ratio on the rheological properties of CEAC. Figure 13a shows the fitting relationship between the shear stress and shear rate of CEAC at different sand–binder ratios. The results showed that there was a good linear relationship between the shear rate and shear stress, and R^2^ was between 0.92 and 0.99. Figure 13b demonstrates the impact of the S/B on the rheological parameters of CEAC. As the S/B increased, both the yield stress and plastic viscosity of CEAC rose. This trend primarily stems from the enhanced sand content, which elevated the likelihood of particle contact and collision, thereby boosting the yield stress. Additionally, the augmented sand content resulted in a thinner coating of gelling material on the sand particle surfaces, increasing interparticle friction and subsequently leading to higher plastic viscosity [29]. Considering the combined effect of the S/B on CEAC spraying performance, an optimal S/B of 0.5 was established, at which point the yield stress and plastic viscosity were measured as 94.94 Pa and 2.98 Pa·s, respectively.

#### 3.1.3. Effect of the Substitution Rate of Calcium Aluminate Cement(CAC)

**Figure 14:**
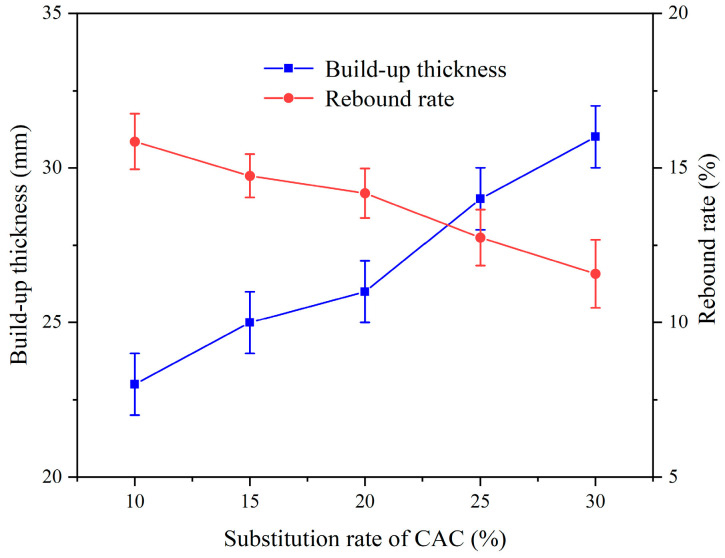
Effect of the CAC substitution rate on the spraying performance of CEAC.

Figure 14 shows the effect of the substitution rate of CAC on the spraying performance of CEAC, and it can be seen that with the increase in the substitution rate of CAC, the build-up thickness of CEAC continued to increase, and the rebound rate continued to decrease. This was because, compared with ordinary Portland cement, CAC had a higher chemical reaction activity, which could cause hydration and hardening reactions to occur more quickly, thus forming a compact structure [30]. The best CEAC spraying performance was achieved when the substitution rate of the CAC was 30%.

**Figure 15:**
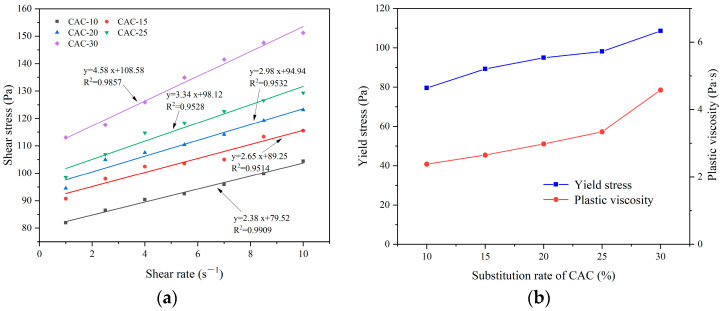
Effect of the CAC substitution rate on the rheological parameters of CEAC: (**a**) rheological parameter fitting curve and (**b**) yield stress and plastic viscosity.

Figure 15 shows the effect of the CAC substitution rate on the rheological properties of CEAC. Figure 15a shows that there was a good linear relationship between shear rate and shear stress at different CAC substitution rates, and R^2^ was between 0.95 and 0.99. Figure 15b shows the effect of the substitution rate of CAC on the rheological parameters of the CEAC. From Figure 15b, it can be seen that the yield stress and plastic viscosity of CEAC increased as the substitution rate of CAC increased. This was because the hydration process of CAC released a large amount of heat; it accelerated the hydration process of the whole cement system in a negative-temperature environment, which ultimately led to an increase in yield stress and plastic viscosity [31]. To ensure that the CEAC had good spraying performance and working performance at the same time, the optimal substitution rate of CAC was determined to be 25%. At this time, the build-up thickness and rebound rate of CEAC after one spraying treatment were 29 mm and 12.75%, respectively, and the yield stress and plastic viscosity were 98.12 Pa and 3.34 Pa·s, respectively.

#### 3.1.4. Effect of the Emulsified Asphalt Content

**Figure 16:**
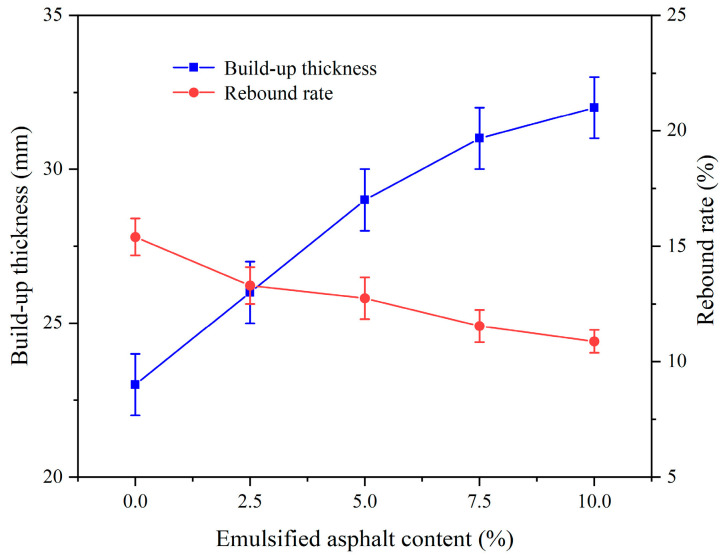
Effect of the emulsified asphalt content on the spraying performance of CEAC.

Figure 16 illustrates the influence of emulsified asphalt content on the spraying efficiency of CEAC. It can be observed that as the emulsified asphalt content increased, there was a gradual enhancement in the build-up thickness during the spraying process of CEAC, while the rebound rate experienced a gradual decline. When the content of emulsified asphalt was 10%, the CEAC spraying performance was the best. This was because the hydration process of cement broke the double electron layer structure in emulsified asphalt, thus accelerating its demulsification, which in turn led to the destabilization and dehydration of the emulsified asphalt, forming asphalt particles with a certain viscosity [32]. When CEAC was sprayed onto a wall, the moisture in the material detached due to its impact on the wall, and the asphalt particles aggregated with each other due to extrusion so that the overall cohesion increased, thus improving the build-up thickness and lowering the rebound rate.

**Figure 17:**
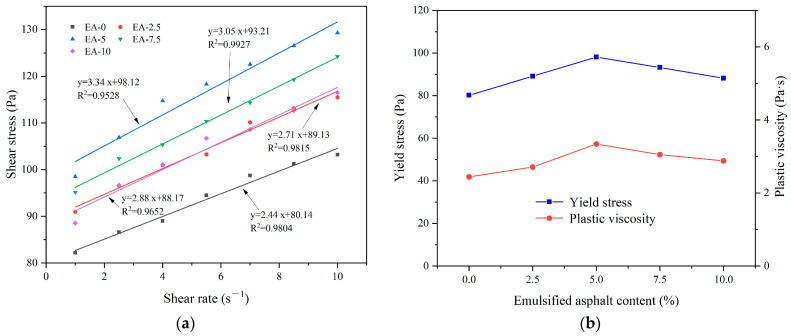
Effect of the emulsified asphalt content on the rheological parameters of CEAC: (**a**) rheological parameter fitting curve and (**b**) yield stress and plastic viscosity.

Figure 17 shows the effect of emulsified asphalt content on the rheological properties of CEAC. Figure 17a shows that there was a good linear relationship between shear rate and shear stress at different emulsified asphalt contents, and R^2^ was between 0.95 and 0.99. Figure 17b shows the effect of the emulsified asphalt content on the rheological parameters of the CEAC. With increasing emulsified asphalt content, the yield stress and plastic viscosity of CEAC first increased and then decreased, and the maximum value occurred when the content of emulsified asphalt was 5%. This was because in CEAC, the hydration process of the cement and the breaking process of the asphalt emulsion were carried out simultaneously. When the emulsified asphalt content fell below 5%, the hydration of cement dominated, while the emulsification of asphalt particles resulted in an elevation of the overall system’s viscosity. This viscosity increase led to greater friction among particles, subsequently boosting the yield stress and plastic viscosity of the CEAC. When the content of emulsified asphalt was greater than 5%, the emulsification of asphalt dominated, and too many asphalt particles were adsorbed onto the cement particles, thus delaying the hydration and hardening of the cement [33]. In addition, the emulsifier component in emulsified asphalt was a surfactant, which was similar to the water reducer component and had a lubricating effect, thus reducing the yield stress and plastic viscosity of CEAC [34]. Combined with the good rheological properties of CEAC when the emulsified asphalt content was 5%, the optimal content of emulsified asphalt was determined to be 5%, at which time the build-up thickness and rebound rate of CEAC after one spraying treatment were 29 mm and 12.75%, respectively, and the yield stress and plastic viscosity were 98.12 Pa and 3.34 Pa·s, respectively.

#### 3.1.5. Effect of PVA Content

**Figure 18:**
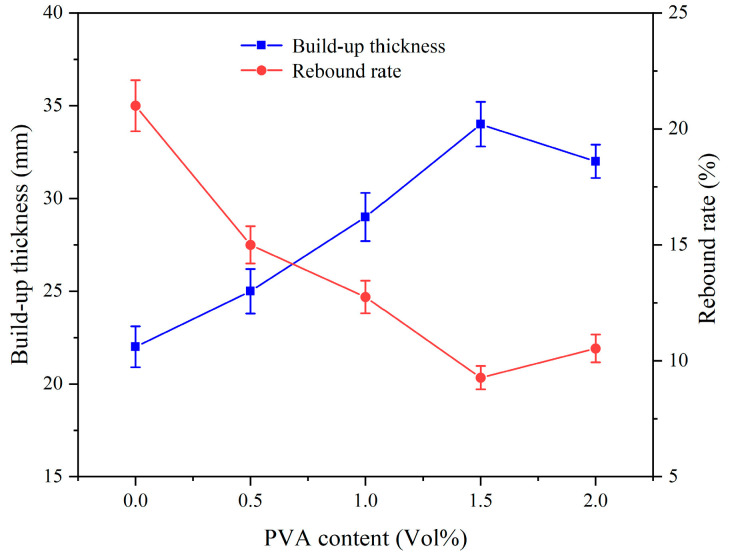
Effect of the PVA content on the spraying performance of CEAC.

Figure 18 illustrates the impact of PVA content on the spraying performance of CEAC. As the PVA content increased, the CEAC’s build-up thickness initially rose and then gradually declined. Alternately, the rebound rate initially decreased, but later on, it exhibited a rise. This was due to the friction created by the disordered fiber distribution within the sprayed cement-based material, which interfered with the slurry’s flow, decreasing its fluidity while enhancing stability [35]. Furthermore, as PVA fibers are hydrophilic, they absorbed water molecules or attracted them to their surface, which not only accelerated cement hardening but also increased slurry viscosity [36]. However, when the content of PVA exceeded 1.5%, the mixing of CEAC slurry was uneven, resulting in poor fluidity and a decrease in the spraying performance of CEAC [37].

**Figure 19:**
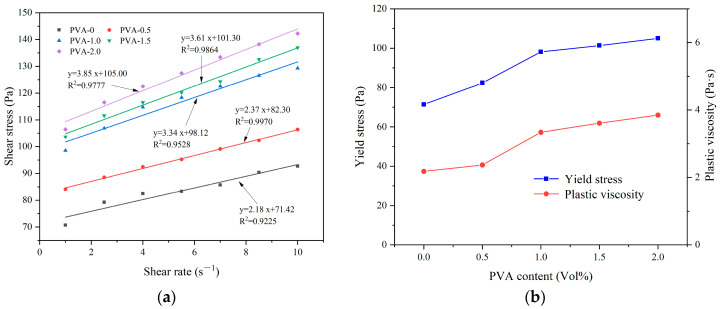
Effect of PVA content on the rheological parameters of CEAC: (**a**) rheological parameter fitting curve and (**b**) yield stress and plastic viscosity.

Figure 19 shows the effect of PVA content on the rheological properties of CEAC. Figure 19a shows that there was a good linear relationship between shear rate and shear stress at different PVA contents, and R^2^ was between 0.92 and 0.99. Figure 19b demonstrates the influence of PVA content on the rheological parameters of CEAC. As the PVA content rose, both the yield stress and plastic viscosity of CEAC increased. This trend was attributed to the water absorption properties of PVA, which reduced the thickness of the water film surrounding solid particles. Consequently, the likelihood of collisions and friction among solid particles intensified. Under these conditions, the yield stress and plastic viscosity of CEAC were enhanced [38]. Given that CEAC exhibited optimal spraying performance when the PVA content was 1.5%, we determined this to be the ideal PVA content. At this point, the build-up thickness and rebound rate of CEAC after one spraying treatment were 34 mm and 9.27%, respectively, and the yield stress and plastic viscosity of CEAC were measured to be 101.30 Pa and 3.61 Pa·s, respectively.

### 3.2. Correlation between Spraying Performance and Rheological Parameters

**Figure 20:**
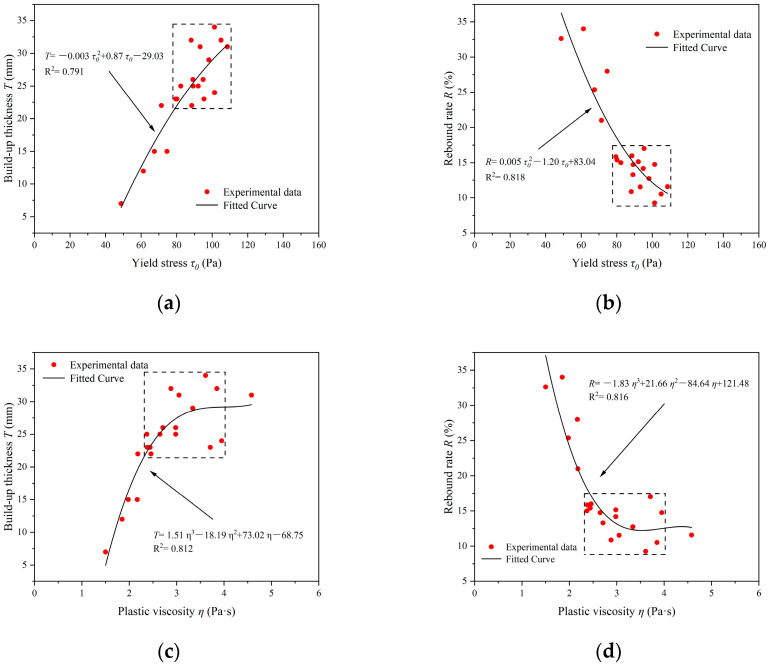
The relationship between spraying performance and rheological parameters of CEAC: (**a**) Yield stress and build-up thickness; (**b**) Yield stress and rebound rate; (**c**) Plastic viscosity and build-up thickness and (**d**) Plastic viscosity and rebound rate.

Figure 20 shows the correlation between the spraying performance and rheological parameters of CEAC. As depicted in Figure 20a,c, both the yield stress (*τ*_0_) and plastic viscosity (*η*) of CEAC exhibited a positive association with the build-up thickness (*T*) achieved during a single spraying event. The specific equations governing these relationships are presented in Equations (3) and (4):

(3) $$T=-0.003\tau_{0}^{2}+0.87\tau_{0}-29.03$$

(4) $$T=1.51\eta^{3}-18.19\eta^{2}+73.02\eta -68.75$$

As depicted in Figure 20b,d, both the *τ*_0_ and *η* of CEAC exhibited a negative correlation with the rebound rate ©. The specific equations that describe these relationships were provided in Equations (5) and (6):

(5) $$R=0.005\tau_{0}^{2}-1.20\tau_{0}+83.04$$

(6) $$R=-1.83\eta^{3}+21.66\eta^{2}-84.64\eta +121.48$$

It is well known that as the *τ*_0_ increases, the material becomes less fluid. When the *τ*_0_ of CEAC increases, its *T* increases, and the *R* decreases. As the *η* rose, the cohesive force of the material was also enhanced, leading to an increase in *T* and a corresponding decrease in *R*. From Figure 15d and Figure 20c, it can be seen that there is a clear turning point in plastic viscosity. When the plastic viscosity was near this turning point, CEAC had good spraying performance, and the influence of plastic viscosity on spraying performance was not significant. Figure 20 illustrates that CEAC exhibited superior spraying performance when the *τ*_0_ fell within the range of 79.52–108.58 Pa and the *η* lay between 2.37 and 3.95 Pa·s. Within this range, the *T* varied from 22 to 34 mm, while the *R* remained between 9.27 and 17.01%.

**Figure 21:**
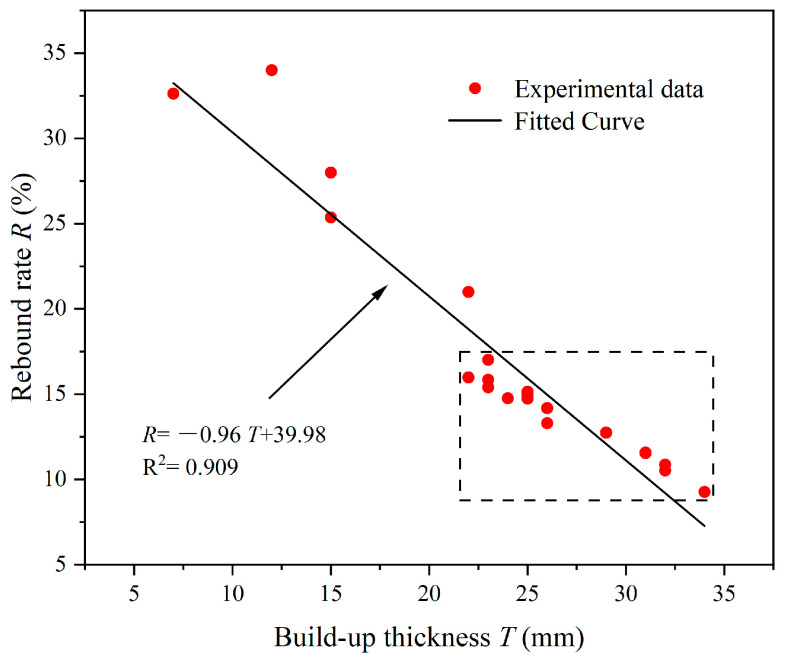
Relationship between the *R* and *T* of CEAC.

Figure 21 shows the relationship between the *R* and *T* of the CEAC. The *T* of the CEAC was linearly and negatively correlated with the *R*, and the fitting relationship is shown in Equation (7). Yun et al. [19] also obtained a similar conclusion in their study.

(7) R=−0.96T+39.98

### 3.3. Frost Resistance

After analyzing the spraying performance and rheological characteristics of CEAC, we have arrived at an optimal mixing ratio. This includes a water–solid ratio of 0.14, a sand–binder ratio of 0.5, a 25% substitution rate for calcium aluminate cement, a 5% emulsified asphalt content, and a 1.5% volumetric content of PVA fibers. With these settings, the build-up thickness and rebound rate were recorded as 34 mm and 9.27%, respectively, while the yield stress and plastic viscosity measured 101.30 Pa and 3.61 Pa·s, respectively. Using this optimal mix ratio for CEAC, we further investigated the frost resistance of both the cement slurry (CS) without emulsified asphalt and CEAC.

**Figure 22:**
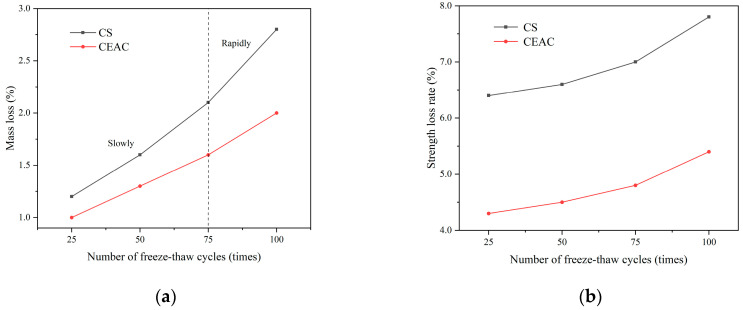
Frost resistance test results of CS and CEAC: (**a**) mass loss and (**b**) strength loss rate.

Figure 22a shows the variation curves of the mass loss rates of CS and CEAC under the action of freeze–thaw cycles. The mass loss rates of CS and CEAC increased continuously with an increasing number of freeze–thaw cycles. Under the same number of freeze–thaw cycles, the mass loss rate of the CEAC was lower than that of the CS. The mass loss rate after 100 freeze–thaw cycles was only 2.0%. This was because, in CEAC, the emulsified asphalt could enhance the adhesion between aggregates after emulsification, which improved the integrity of the specimen; on the other hand, the water content of the specimen was reduced due to the water barrier of the asphalt content, which reduced the probability of damage to the microcellular freezing under freeze–thaw cycles. In addition, the mass loss rates of CS and CEAC first increased slowly and then increased rapidly, showing similar patterns of change. This was because during the pre-freeze–thaw cycle period, the freeze–thaw damage only occurred on the specimen surface. After 75 freeze–thaw cycles, the rate of mass loss increased significantly due to the continuous action of freeze–thaw cycles, which led to the intensification of matrix damage [15,39].

Figure 22b shows the varying trends of the strength loss rates for both CS and CEAC as they undergo freeze–thaw cycles. It is evident that as the number of freeze–thaw cycles increased, the strength loss rates for both CS and CEAC continued to rise. Under the same number of freeze–thaw cycles, the strength loss rate of the CEAC was lower than that of the CS. On the one hand, the emulsifier component of emulsified asphalt was a surfactant, similar to air-entraining agents, which could introduce stable and small bubbles in concrete to isolate water transport channels, thus relieving water crystallization pressure and inhibiting the formation and growth of ice crystals [40]. On the other hand, due to the simultaneous destruction of the emulsified asphalt emulsion and the cement hydration process, the generated asphalt film and cement hydration products intertwined with the flocculent colloid, and this flocculent colloid filled up the specimen inside some large pores, thereby inhibiting the damage of the specimen during freeze–thaw cycling. In summary, the frost resistance of CEAC was superior to that of CS.

### 3.4. Hole Structure Analysis

**Figure 23:**
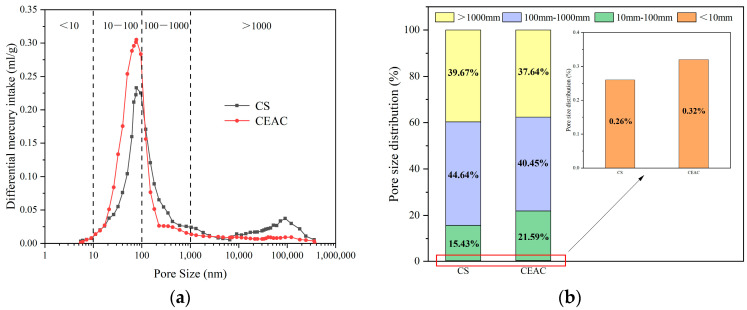
MIP test results of CS and CEAC: (**a**) pore size differential curve and (**b**) pore size distribution.

Based on the findings reported by Odler et al. [41], cement concrete exhibits four distinct pore size categories: gel pores (with diameters below 10 nm), transition pores (spanning from 10 to 100 nm), capillary pores (ranging from 100 to 1000 nm), and macropores (exceeding 1000 nm in diameter). Furthermore, the pore structures of CS and CEAC were analyzed through the MIP test, and the outcomes of this test are presented in Figure 23. Figure 23 shows that the volume of capillary pores and macropores in CS reached 44.64% and 39.67%, respectively, of the total pore volume, while the volume of capillary pores and macropores in CEAC reached 40.45% and 37.64%, respectively, of the total pore volume. Hover et al. [42] concluded that capillary pores and macropores mainly affect the frost resistance of concrete. Therefore, the lower capillary pore content and macropore content corroborate that CEAC had better frost resistance.

The above analysis revealed that the emulsified asphalt and cement cemented to each other, generated a flocculent colloid, and spread to the surrounding space to effectively fill the large pores, thus reducing the proportion of large pores. Therefore, mixing emulsified asphalt in cement-based spraying materials can effectively improve the shortcomings of high porosity and poor densification, thus improving frost resistance.

## 4. Conclusions

This study comprehensively explores the impact of various factors, including water–solid ratio, sand–binder ratio, calcium aluminate cement substitution rate, emulsified asphalt dosing, and PVA fiber content, on the spraying performance and rheological properties of CEAC. The relationship between spraying performance and rheological parameters was established, and the optimum mixing ratio of CEAC was obtained. In addition, the frost resistance of the cement slurry (CS) without emulsified asphalt and with CEAC was investigated, and the pore structure characteristics of the materials were analyzed via a MIP test. The following conclusions were obtained:

(1) Based on the spray performance and rheological parameters, the optimal mix ratio for CEAC was determined using the control variable method: a water–solid ratio of 0.14, a sand–binder ratio of 0.5, a calcium aluminate cement substitution rate of 25%, an emulsified asphalt dosage of 5%, and a PVA fiber volume content of 1.5%.
(2) Utilizing the correlation between spraying performance and rheological parameters, this study determined the ranges of spraying performance and rheological parameters for CEAC suitable for spraying in cold environments. Specifically, the yield stress ranged from 79.52 to 108.58 Pa, the plastic viscosity varied between 2.37 and 3.95 Pa·s, the build-up thickness was between 22 and 34 mm, and the rebound rate ranged from 9.27 to 17.01%. These ranges provide valuable guidance for the application of CEAC spraying in negative-temperature environments.
(3) The build-up thickness of CEAC was positively correlated with yield stress and plastic viscosity, and the rebound rate was negatively correlated with yield stress and plastic viscosity. The rheological performance parameters of CEAC can be used to characterize its spraying performance within a specific range.
(4) The results of frost resistance testing conducted on CS and CEAC, both prepared using the optimal mix ratio for CEAC spraying in cold environments, revealed that CEAC exhibited lower mass loss and strength loss rates compared to CS after an equal number of freezing and thawing cycles. This indicates that CEAC possesses superior frost resistance properties compared to CS.
(5) The results of the MIP test show that the content of capillary pores and macropores mainly affects the freezing resistance of CEAC, and the smaller their content was, the better the freezing resistance of CEAC was.

## Figures and Tables

**Table 1:** Composition of the cement.

Type of Cement	Mass Fraction (wt.%)									
	SiO_2_	CaO	Al_2_O_3_	SO_3_	MgO	Fe_2_O_3_	KO_2_	Na_2_O	TiO_2_	LOI
Portland cement	21.50	64.20	4.14	2.89	2.57	2.40	0.84	0.67	0.32	0.40
Calcium aluminate cement	0.68	26.80	71.30	0.04	0.46	0.09	0.02	0.37	0.11	0.07

**Table 2:** Performance indices of PVA fiber.

Diameter (μm)	Length (mm)	Tensile Strength (MPa)	Young’s Modulus (Gpa)	Elongation (%)	Density (g/cm^3^)
31	6	1500	40	6	1.28

**Table 3:** Test mix ratio of CEAC.

Group	W/S	S/B	CAC (%)	EA (%)	PVA (%)
W/S-0.12	0.12	0.4	20	5	1.0
W/S-0.13	0.13				
W/S-0.14	0.14				
W/S-0.15	0.15				
W/S-0.16	0.16				
S/B-0.2	0.14	0.2	20	5	1.0
S/B-0.3		0.3			
S/B-0.4		0.4			
S/B-0.5		0.5			
S/B-0.6		0.6			
CAC-10	0.14	0.5	10	5	1.0
CAC-15			15		
CAC-20			20		
CAC-25			25		
CAC-30			30		
EA-0.0	0.14	0.5	25	0.0	1.0
EA-2.5				2.5	
EA-5.0				5.0	
EA-7.5				7.5	
EA-10.0				10.0	
PVA-0.0	0.14	0.5	25	5	0.0
PVA-0.5					0.5
PVA-1.0					1.0
PVA-1.5					1.5
PVA-2.0					2.0

Note: W/S is the mass ratio of water (sum of water in emulsified asphalt and added water) to solids (sum of cement, sand, and emulsified asphalt solids); S/B is the mass ratio of sand to binder (sum of cement and emulsified asphalt solids); CAC is the mass percentage of calcium aluminate cement to total cement; EA is the mass percentage of emulsified asphalt solids to binder; PVA is the volume of fibers to slurry as a percentage.

## Data Availability

Some or all data, models, or code that support the findings of this study are available from the corresponding author upon reasonable request.
